# Diagnostic proficiency and reporting of Lassa fever by physicians in Osun State of Nigeria

**DOI:** 10.1186/1471-2334-14-344

**Published:** 2014-06-20

**Authors:** Samuel Anu Olowookere, Akinola Ayoola Fatiregun, Olalere Omoyosola Gbolahan, Ebenezer Gbenga Adepoju

**Affiliations:** 1 Department of Community Health, Faculty of Clinical Sciences, College of Health Sciences, Obafemi Awolowo University, Ile-Ife, Osun state, Nigeria; 2 Department of Epidemiology and Medical Statistics, University of Ibadan, Ibadan, Nigeria; 3 Department of Maxillofacial Surgery, University of Ibadan, Ibadan, Nigeria; 4 Department of Preventive Medicine, State Specialist Hospital, Asubiaro, Osogbo, Osun-State, Nigeria

**Keywords:** Assessment, Diagnosis, Reporting, Lassa fever, Nigerian physicians

## Abstract

**Background:**

Lassa fever is highly contagious and commonly results in death. It is therefore necessary to diagnose and report any suspected case of Lassa fever to facilitate preventive strategies. This study assessed the preparedness of physicians in the diagnosis and reporting of Lassa fever.

**Methods:**

The study design was descriptive cross-sectional. The consenting medical doctors completed a self-administered questionnaire on the diagnosis and reporting of Lassa fever. Descriptive and inferential statistics were used in data analyses.

**Results:**

One hundred seventy-five physicians participated in the study. The mean age was 41.5 ± 10.9 years (range, 24–75 years). Most of the physicians were male (78.9%) and had practiced medicine ≥ 20 years (51.5%). Most of the physicians had a good knowledge regarding the diagnosis and reporting of Lassa fever; however, none of the physicians had ever diagnosed or reported a suspected case. Predictors of good knowledge include male sex, not practicing at a secondary health care level and post graduation year more than 20 years.

**Conclusion:**

There is disparity in knowledge and practices of physicians regarding the diagnosis and reporting of Lassa fever. Thus, it is necessary to improve the knowledge and practices of physicians regarding the diagnosis and reporting of Lassa fever.

## Background

Lassa fever is a viral haemorrhagic fever caused by a rodent-borne arenavirus that is endemic to West Africa [1-3]. Arenaviruses are emerging in the African continent and can cause haemorrhagic fevers with case fatalities between 10% and 20% [4,5]. These viruses are mainly transmitted through contact with the excreta of the natural host (rodents of the family Muridae) [6-8]. The Old World arenavirus, Lassa virus, causes up to 300,000 cases of Lassa fever annually in West Africa [9-11]. In the 1970s the rodent host of Lassa virus was classified as *Mastomys natalensis*. Other rodent hosts, such as *M. erythroleucus*, and the Rattus and Mus genera, have been suggested [12-14]. Health care workers are predominantly infected and likely to die from Lassa fever in the central and southern parts of Nigeria, most often due to poor medical practices, late diagnosis, and treatment [10,15]. The establishment of diagnostic facilities that can provide rapid molecular testing at referral centers in the disease-endemic zones has been suggested as a partial solution to this problem. This testing would facilitate appropriate case and contact management, including early treatment and post-exposure prophylaxis with Ribavirin, and eventually raise awareness that Lassa fever should be considered in every severe febrile illness in these regions of Nigeria and West Africa [14,16].

Although no previous cases of Lassa fever has been reported in the State of Osun in Nigeria, outbreaks of the disease were reported in several states of Nigeria including nearby Edo state.

In the first quarter of 2012, 623 suspected cases, including 70 deaths were recorded from 19 of the 36 States of Nigeria. Laboratory analysis undertaken at the Irrua Specialist Teaching Hospital, Irrua Edo State, Nigeria confirmed the presence of Lassa virus infection in 108 patients. Three doctors and four nurses were among the fatalities [17].

Since medical doctors are the first point of contact in diagnosing and reporting cases of Lassa fever, it is imperative to conduct baseline studies to assess the diagnostic knowledge and reporting of Lassa fever among physicians in Nigeria, hence the need for this study.

This study determined the preparedness of physicians in the diagnosis and reporting of Lassa fever.

## Methods

### Study setting

Osun state is one of 36 states in Nigeria, and is located in the southwestern part of Nigeria. Osun state is bounded on the north by Kwara state, on the east by Ekiti and Ondo states, on the south by Ogun state, and on the west by Oyo state. Osun state has 30 local government areas with several maternity centres, comprehensive health centres, and state hospitals spread across the state. Also, LAUTECH Teaching Hospital, Osogbo and Obafemi Awolowo University Teaching Hospital Complex, Ile-Ife are situated in Osun state. As at the time this study was conducted, about 600 medical doctors practice medicine in Osun state.

### Study design

This was a descriptive cross-sectional study conducted in the month of June, 2013. All physicians attending a Nigerian Medical Association (NMA)-organized Continuing Medical Education programme at the NMA house in Osogbo, which is a requirement for annual licensure, were approached, after taken their consent.

### Data collection

A validated (face validity) semi-structured, self-administered questionnaire was used to obtain data on the respondents sociodemographic characteristics, knowledge, diagnosis, management and reporting of Lassa fever.

### Data analysis

The data obtained were entered into SPSS version 16 and analyzed as frequencies for categorical variables, and mean and standard deviation for continuous variables. Knowledge score was computed for a 19-item question on general knowledge of Lassa fever. Each item was assigned ‘+1’ for correct knowledge and ‘0’ for incorrect knowledge. The knowledge score was graded as good or appropriate (if respondent scored ≥ 10 points), and not good or not appropriate (if score was < 10 points). Knowledge score was also computed for a 10-item question on Lassa fever management and reporting. Each item was assigned ‘+1’ for correct knowledge and ‘0’ for incorrect knowledge. The score was classified into good or appropriate (if respondent scored > 5 points) and not good or not appropriate (≤5 points). Bivariate chi-square test and multivariate logistic analyses were performed on respondent’s characteristics and general knowledge on Lassa fever, as well as knowledge on management and reporting. Variables in the bivariate test with p-value of <0.2 were included in the multi-variate model. A p value <0.05 was accepted as significant.

### Ethical clearance

Permission to conduct the study was sought from the Osun state branch of the Nigerian Medical Association, while verbal consent was obtained from every medical doctor approached to participate in the study. Permission to conduct the study was granted by the State Hospital Ethics and Research Committee (protocol no: SHO/ERC/13/007). The data collected was entered and kept in a password-protected computer.

## Results

### Characteristics of respondents

Out of 192 physicians, 5 declined participation while 9 did not return their questionnaire. Three questionnaires were excluded from analysis because of non-completeness. One hundred seventy-five questionnaires with completed data were analysed. The mean age of the study participants was 41.5 ± 10.9 years, majority (78.9%) of them were males, 83.4% were married, while a larger proportion (92.6%) were of Yoruba tribe. More than half (51.5%) of the study participants had a post University graduation of more than 20 years to the time of the data collection, 47.3% ranged between 10 to 20 years and only 1.2% had graduated less than 10 years ago (Table 1). About 44.9% of the participants had more than five years of post residency training. The mean number of years of participant’s clinical practice in primary, secondary and tertiary health care was 6.3, 8.8, and 6.5 years respectively.

**Table 1:** Respondent’s characteristics

**Variable**	**Frequency (n)**	**Percentage (%)**
Age:		
20–24 years	2	1.4
25–29 years	18	12.2
30–34 years	26	17.6
35–39 years	30	20.3
≥ 40 years	72	48.6
Gender:		
Male	138	78.9
Female	37	21.1
Ethnicity:		
Yoruba	162	92.6
Igbo	8	4.6
Other	5	2.9
Year of graduation:		
More than 20 years	85	51.5
10–20 years	78	47.3
Less than 20 years	2	1.2
Years of post residency:		
≤ 5 years	27	55.1
> 5 years	22	44.9
Primary clinical practice:		
Yes	70	40
No	105	60
Secondary clinical practice:		
Yes	111	63.4
No	64	36.6
Tertiary clinical practice:		
Yes	99	56.6
No	76	43.4
Designation:		
Consultant	33	18.9
Medical officer	29	16.6
Other	22	12.6
Senior medical officer	21	12.0
Registrar	19	10.9
Chief medical officer	20	11.4
Senior registrar	14	8.0
House officer	10	5.7
Principal medical officer	7	4.0
Department:		
A & E	3	2.4
Anesthesia	3	2.4
Biochemistry	2	1.6
Dental health	16	12.8
Community health	11	8.8
ENT	2	1.6
Family medicine	7	5.6
GOPD	28	22.4
Health centre	9	7.2
Internal medicine	3	2.4
Anatomy	1	0.8
O & G	6	4.8
Ophthalmology	3	2.4
Oral path/OMS	3	2.4
Orthopaedics	1	0.8
Pediatrics	4	3.2
Private practice	3	2.4
Psychiatry	1	0.8
Radiology	1	0.8
Surgery	17	13.6
Hospital board	1	0.8

About 18.9% of the respondents were consultants, only 16.6% were medical officers, and the rest are shown in Table 1. Some physicians worked in primary care such as General out-patient department (GOPD) (22.4%), Family medicine (5.6%), Community Health (8.8%), Health centre (7.2%) and private practice (2.4%).

### Lassa fever knowledge

**Figure 1:**
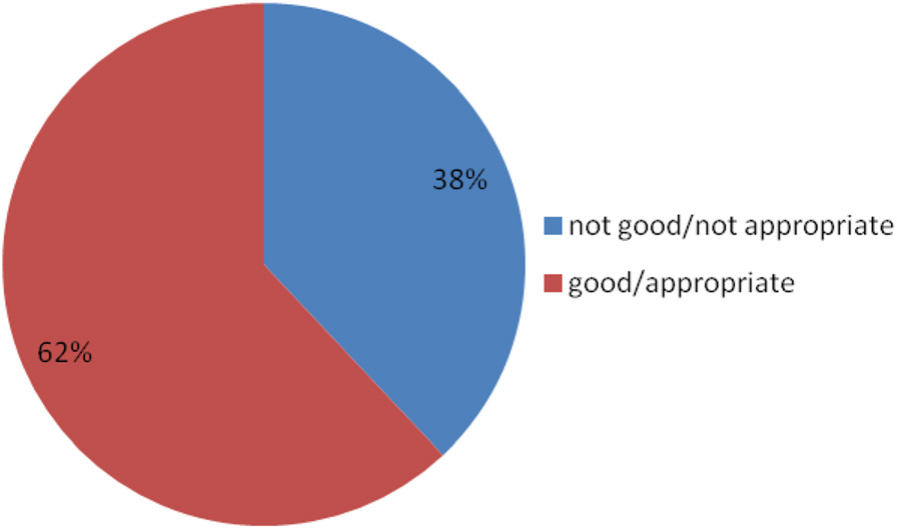
Graded score on Lassa fever knowledge.

Figure 1 shows that more than half (62%) of the respondents had good general knowledge on Lassa fever. As shown in Table 2, majority (93.1%) of the respondents knew that Lassa fever is a viral infection, 57.1% agreed that the incubation period ranges from 1 – 3 weeks, and 85.2% agreed the infection can be characterized by fever > 38°C. Majority (82.9%) of the respondents reported that cases can not be confirmed without laboratory diagnosis, and 98.2% of them disagreed to ‘pediatrics clients being excluded from diagnoses’. Only 34.8% of the respondents agreed that aerosol transmission of the infection is possible, while 43.0% reported semen of case being capable of transmitting infection.

**Table 2:** Lassa fever knowledge

**Variable**	**Frequency (n)**	**Percentage (%)**
Lassa fever is a viral disease:		
Yes	163	93.1
No	5	2.9
Incubation period ranges for 1–3 weeks:		
Yes	100	57.1
No	64	36.6
Reservoir – rat:		
Yes	169	96.6
No	3	1.7
Transmitted by rat biting:		
Yes	34	19.4
No	131	74.9
Cases are characterized by fever >38°C:		
Yes	144	85.2
No	25	14.8
Retrosternal pain is a common symptom:		
Yes	66	41.5
No	93	58.5
Bleeding from mucosal surface is a sign:		
Yes	142	84.5
No	26	15.5
Vomiting and shock is rarely observed:		
Yes	43	25.3
No	127	74.7
Proteinuria is suggestive:		
Yes	44	27.2
No	127	72.8
Fever refractory to treat and mucosal bleeding is a sign:		
Yes	134	79.3
No	35	20.7
Absence of profuse mucosal bleeding exclude diagnosis:		
Yes	5	3
No	161	97
Pediatrics clients are excluded from diagnosis:		
Yes	3	1.8
No	164	98.2
Cases can be confirmed without lab diagnosis:		
Yes	28	17.1
No	136	82.9
Aerosol transmission:		
Yes	57	34.8
No	107	65.2
Causative organism penetrates unbroken skin:		
Yes	37	22.8
No	125	77.2
Cases cease to be infectious after acute phase:		
Yes	18	11.0
No	146	89.0
Semen of case is capable of transmitting infection:		
Yes	71	43.0
No	94	57.0
Jaundice is a common manifestation:		
Yes	103	62.4
No	62	37.6
Bodies of dead cases constitute potential harm:		
Yes	122	73.1
No	45	26.9

### Knowledge on Lassa fever treatment

Table 3 shows the response of the participants to knowledge of Lassa fever treatment. Ribavirin was reported by 80.6% of the respondents as the drug used for treatment of Lassa fever, other drugs listed were Corticosteroid (5.6%), Ranitidine (5.6%), and antiviral drug (8.2%). Majority (61.2%) of the respondents reported absence of bleeding to be a good prognostic sign during Lassa fever treatment.

**Table 3:** Treatment agent

**Variable**	**Frequency (n)**	**Percentage (%)**
Drug used for treatment:		
Ribavirin	29	80.6
Corticosteroid	2	5.6
Ranitidine	2	5.6
Antiviral drug	3	8.2
Patients nursed in open wards:		
Yes	**7**	4.5
No	133	84.7
Not sure	17	10.8
Corticosteroids important in management:		
Yes	53	34.2
No	31	20.0
Not sure	71	45.8
Absence of bleeding good prognostic sign:		
Yes	98	61.2
No	16	10.0
Not sure	46	28.8

### Knowledge on Lassa fever reporting

Few (19.4%) of the respondents agreed that a critical number of cases must be observed before reporting, while 80.6% did not agree to that. Most (82.5%) of the participants agreed that suspected cases qualify for reporting, while 73% agreed that confirmed cases can be reported. More than half (61.1%) of the respondents reported that it is not true that cases should be reported weekly for administrative efficiency (Table 4).

**Table 4:** Knowledge on Lassa fever reporting

**Variable**	**Frequency (n)**	**Percentage (%)**
Critical minimum number of cases must be observed before reporting:		
True	30	19.4
Not true	125	80.6
Suspected case:		
True	138	82.5
Not true	28	17.5
Confirmed case:		
True	43	27
Not true	116	73
Weekly reporting:		
True	58	38.9
Not true	91	61.1
Federal:		
True	79	50
Not true	79	50
State:		
True	83	52.5
Not true	75	47.5
Local:		
True	67	43.8
Not true	86	56.2

### Respondent’s characteristics and Lassa fever knowledge

Table 5 shows the association of respondent’s characteristics with knowledge of Lassa fever. A higher proportion (77.3%) of the respondents who were older than 40 years had an appropriate knowledge on Lassa fever, compared to 21.9% of those who were younger than 40 years, although this was not significant (p = 1.000). Surprisingly, a significantly higher proportion (35.8%) of the respondents who did not have clinical practice in a secondary health facility had an appropriate knowledge on Lassa fever, compared to 19.8% of those who were had practiced in a secondary health facility (p = 0.049).

**Table 5:** Association between respondent’s characteristics and Lassa fever knowledge

	**Knowledge**			
**Variable**	**Appropriate**	**Not appropriate**	**χ**^**2**^	**P-value**
Age:				
< 40 years	14 (21.9)	50 (78.1)		
≥ 40 years	51 (77.3)	15 (22.7)	0.907	1.000
Gender:				
Male	31 (25.8)	89 (74.2)		
Female	22 (75.9)	7 (24.1)	0.851	1.000
Ethnicity:				
Yoruba	34 (24.6)	104 (75.4)		
Igbo	3 (37.5)	5 (62.5)		
Other	1 (33.3)	2 (66.7)	0.757	0.685
Marital status:				
Currently married	34 (26.6)	94 (73.4)		
Currently not married	4 (19.0)	17 (81.0)	0.414	0.594
Years of graduation:				
More than 20 years	20 (26.7)	55 (73.3)		
10–20 years	16 (23.2)	53 (76.8)	0.865	0.471
Less than 10 years				
Years of residency:				
≤ 5 years	6 (27.3)	16 (72.7)		
> 5 years	5 (25.0)	15 (75.0)	0.867	1.000
Primary health care				
practice: Yes	13 (20)	52 (80)		
No	25 (29.8)	59 (70.2)	0.175	0.190
Secondary health care				
practice: Yes	19 (19.8)	77 (80.2)		
No	19 (35.8)	34 (64.2)	0.031	0.049
Tertiary health care				
practice: Yes	23 (26.7)	63 (73.3)		
No	15 (23.8)	48 (76.2)	0.685	0.708

### Predictors of knowledge on Lassa fever

Table 6 shows the result of the multivariable model. Respondents who were not practicing at a secondary health care level were more likely to have an appropriate knowledge on Lassa fever (OR = 0.41, 95% CI = 0.192–0.889).

**Table 6:** Logistic regression for respondent’s characteristics and Lassa fever knowledge

**Variable**	**OR**	**95% CI**	**P-value**
Primary health care practice	0.54	0.245–1.180	0.122
Secondary health care practice	0.41	0.192–0.889	0.024

### Association between respondent’s characteristics and knowledge on management and reporting of Lassa fever

A higher proportion (42.6%) of respondents who were males were more likely to have a good knowledge on Lassa fever management and reporting, compared to females (34.3%), p = 0.244. Respondents who had more than 20 years of post graduation from medical school had a higher (49.4%) level of knowledge on Lassa fever management and reporting, compared to those who had less than 20 years of post graduation (33.3%), p = 0.031 (Table 7).

**Table 7:** Association between respondent’s characteristics and knowledge on management and reporting of Lassa fever

	**Knowledge**			
**Variable**	**Good**	**Fair**	**χ**^**2**^	**p-value**
Age:				
< 40 years	29 (40.3)	43 (59.7)		
≥ 40 years	30 (44.1)	38 (55.9)	0.211	0.386
Gender:				
Male	55 (42.6)	89 (74.2)		
Female	12 (34.3)	7 (24.1)	0.794	0.244
Ethnicity:				
Yoruba	59 (39.1)	92 (60.9)		
Igbo	4 (50.0)	4 (50.0)		
Other	4 (80.0)	1 (20.0)	3.646	0.162
Marital status:				
Currently married	59 (42.1)	81 (57.9)		
Currently not married	4 (33.3)	16 (66.7)	0.658	0.281
Years of graduation:				
> 20 years	40 (49.4)	41 (50.6)		
≤ 20 years	25 (33.3)	50 (66.7)	4.127	0.031
Years of residency:				
≤ 5 years	10 (41.7)	14 (58.3)		
> 5 years	7 (31.8)	15 (68.2)	0.478	0.351
Primary health care				
practice: Yes	25 (39.1)	39 (60.9)		
No	10 (45.5)	12 (54.5)	0.277	0.389
Secondary health care				
practice: Yes	45 (42.5)	61 (57.5)		
No	2(33.3)	4 (66.7)	0.194	0.503
Tertiary health care				
practice: Yes	38 (40.4)	56 (59.6)		
No	1 (50.0)	1 (50.0)	0.740	0.650

### Predictors of knowledge on Lassa fever management and reporting

In the multi-variate analysis, the only factor that was significantly predictive of knowledge on Lassa fever management and reporting was years of graduation. Table 8 shows that respondents who had post graduation year more than 20 years were more likely to have a good knowledge compared to those who had 20 years or lesser post graduation experience (OR = 0.48, 95% CI = 0.24–0.94).

**Table 8:** Logistic regression for respondent’s characteristics and knowledge on Lassa fever management and reporting

**Variable**	**OR**	**95% CI**	**p-value**
Gender: Male	0.86	0.38 – 1.96	0.72
Female	1		
Ethnicity:			
Yoruba	1		
Igbo	0.65	0.15 – 2.77	0.56
Other	0.13	0.01 – 1.28	0.08
Year of graduation:			
> 20 years	0.48	0.24 – 0.94	0.032
≤ 20 years	1		

## Discussion

This study assessed the knowledge, diagnosis, and reporting of Lassa fever by physicians in Nigeria. Specifically, nearly all respondents had practiced medicine for > 10 years. Despite this, none of the respondents had managed or reported a suspected case of Lassa fever. This was probably because Lassa fever is not one of the differential diagnoses usually considered when patients with febrile illnesses present for evaluation. Several studies on Lassa virus detection have reported that misdiagnoses are common among physicians due to the non-consideration of Lassa fever as a cause of febrile illness and the non-specific clinical signs and symptoms of a large proportion of Lassa infections, combined with the lack of familiarity of physicians with Lassa fever, suggesting that the infection can easily be misdiagnosed [18-22].

However, most physicians interviewed had appropriate knowledge of Lassa fever. Thus, a disconnect between knowledge and practice as probable cases could have presented to these physicians that had been misdiagnosed leading to progression of the disease as seen in areas with low reporting of Lassa fever [21-23]. It is therefore essential that physicians, especially those practicing in primary care, should be targeted in training on emerging diseases to increase their index of suspicion and protect themselves from exposure to this deadly virus. This training will help every physician, whether they have ‘good’ or ‘poor’ knowledge regarding the diagnosis, management, and reporting of Lassa fever. The training should emphasize the concept of universal precautions, which must be observed by every health care worker while interacting with every patient [24-27].

Although all respondents believe the State Ministry of Health and their hospital pharmacy should stock Ribavirin, which is used for post-exposure prophylaxis by all contacts of suspected cases [24,27], none of the physicians could state categorically that Ribavirin is available at all times in the hospitals with which they are affiliated. Also, no physician had the occasion to prescribe the drug previously. This showed the low-risk perception among these physicians of Lassa fever, implying that more efforts should be made by an appropriate authority to make Ribavirin available in all hospitals and equally inform the health care workers for easy access by contacts of suspected cases.

The limitation of this study was that only physicians present at the NMA continuous medical education conference were included. Although, we expected that the programme would require the participation of all doctors, this was not so based on the number who attended. We recognized that selection bias may have occurred as those who attended may be different in some characteristics (which may affect the outcome of the study) from those who did not attend, limiting the generalization of our study findings. However, the type of physicians who see the Lassa fever cases initially are generally primary care physicians, who work in private and public owned health care facilities. These practitioners formed about half (46.4%) of the study participants. Also, the study was cross-sectional and used a self-administered questionnaire. However, to the best of our knowledge, this is the first study to be conducted on the diagnosis and reporting of Lassa fever in the study area. This therefore serves as a baseline study to implement interventions to control possible epidemics of Lassa fever in the study area.

## Conclusion

In conclusion, despite having an appropriate knowledge about Lassa fever, no suspected case has ever been diagnosed or reported by the physician participants. It is necessary to improve physician knowledge and practice on Lassa fever through continuous medical education. It is equally essential to ensure implementation of Lassa fever diagnostics and surveillance.

## Competing interests

The authors declare that they had no competing interests.

## Authors’ contributions

SAO and AAF made substantial contributions to conception and design of the study while all the authors were involved in data collection, analysis and interpretation. All authors were involved in writing the manuscript and approved the final copy.

## Pre-publication history

The pre-publication history for this paper can be accessed here:

http://www.biomedcentral.com/1471-2334/14/344/prepub
